# Supplementary material for: Collaborative intelligence in AI: Evaluating the performance of a council of AIs on the USMLE
Source: PLOS Digit Health. 2025 Oct 9;4(10):e0000787. doi: 10.1371/journal.pdig.0000787 (PMC12510544; doi:10.1371/journal.pdig.0000787)
Supplement: S2 File — (ZIP) [file pdig.0000787.s002.zip › S2 Data Raw data files containing unprocessed question inputs and LLM outputs including transcripts of deliberations/STEP3_Question-2-1_Type-MC-NJ.docx]

**Facilitator** **[Round 1]:** A 34-year-old woman comes to the office because of a 1-month history of worsening right upper quadrant abdominal pain and discomfort. She describes the pain as a dull ache and says it is not affected by eating or defecating. She has not had nausea or changes in appetite or bowel habits. She feels the pain constantly while she is awake, but it rarely keeps her from sleeping. Acetaminophen provides occasional relief. She has been otherwise healthy. Medical history is unremarkable and her only other medication is an oral contraceptive. Vital signs are normal. Abdominal examination discloses hepatomegaly but no palpable masses or evidence of cirrhosis. Results of liver function tests and serum α-fetoprotein concentration are within the reference ranges. Serologic studies for hepatitis B and C are negative. Ultrasonography of the abdomen shows a 4×4-cm mass in the right lobe of the liver. Which of the following is the most likely diagnosis? (A) Hepatic adenoma (B) Hepatocellular cancer (C) Hydatid cyst (D) Metastatic ovarian cancer

**AI_D:** RESTATING THE QUESTION: This clinical scenario revolves around a middle-aged female patient who has been suffering from continuous dull pain in the upper right region of her abdomen, which temporarily lessens with the administration of Acetaminophen. The patient's historical medical records show her health to have been consistent, aside from her current medication that includes an oral contraceptive pill. Clinical examination reveals an enlarged liver, with the absence of any palpable masses or signs of cirrhosis. Standard assessments of her liver function and α-fetoprotein concentration, a marker associated with liver disease, are normal, and viral studies for hepatitis B and C have returned negative. An abdominal ultrasound reveals a 4cm mass within the right lobe of her liver. The question aims to diagnose the most probable cause of these clinical findings. PLANNING: Here, the key assumptions are as follows: (1) the patient's significant medical history is limited to the use of oral contraceptives; (2) the liver function tests and α-fetoprotein concentration are within normal range; (3) Hepatitis B and C are ruled out; (4) ultrasound shows a hepatic mass. Alternately, we could hypothesize that the hepatomegaly and hepatic mass are consequences of her oral contraceptive use, and we need to decide if it could be a benign hepatic adenoma, a malignant hepatocellular carcinoma, a hydatid cyst possibly caused by a parasitic infection, or a metastatic ovarian cancer spread. CLINICAL REASONING: The hepatic adenoma is associated with estrogen use, including oral contraceptives, and typically presents in similar-aged women. Hepatocellular cancer usually presents with elevated α-fetoprotein levels, is often related to cirrhosis, viral hepatitis or chronic alcohol consumption, none of which is present here. Hydatid disease is an infection caused by the Echinococcus tapeworm, often linked to exposure to livestock, which has not been mentioned in the patient's history. While a metastatic ovarian cancer could cause a hepatic mass, it's also less likely due to the lack of accompanying symptoms such as menstrual irregularities, loss of appetite, or unexplained weight loss. CONCLUDING: Therefore, considering the patient’s age, sex, use of oral contraceptives, absence of other risk factors for liver disease, and normal liver function tests and serum α-fetoprotein concentration, 'Hepatic adenoma' is the most likely diagnosis. RESPONSE: The most likely diagnosis in this case is (A) Hepatic adenoma.

**AI_A:** RESTATING THE QUESTION: A 34-year-old woman with no significant medical history presents with a persistent right upper quadrant abdominal pain unaffected by eating or defecating. Physical examination reveals an enlarged liver, but her liver function tests and α-fetoprotein are normal, and she is negative for hepatitis B and C. An abdominal ultrasound shows a mass on her liver. The question requires differentiating between four possible diagnoses: hepatic adenoma, hepatocellular cancer, hydatid cyst, or metastatic ovarian cancer. PLANNING: The assumptions we need to question here focus around determining the type of liver mass. The fact that the patient is taking oral contraceptives, her relatively young age, absence of cirrhosis and the normal α-fetoprotein levels may suggest that the mass could be more on the benign side like a hepatic adenoma. However, given the persistent pain and the homogeneous appearance of the lesion, malignant diagnoses such as hepatocellular cancer or metastatic ovarian cancer cannot be ruled out. Hydatid cyst caused by Echinococcus is also a potential although it is usually seen when exposed to dog feces, or lived or traveled in an endemic area which are not mentioned in the scenario. CLINICAL REASONING: Hepatocellular carcinoma usually arises in the setting of chronic liver disease and cirrhosis, and often presents with an elevated α-fetoprotein level, none of which is seen in this patient. Metastatic ovarian cancer generally presents initially with pelvic symptoms and a mass would likely be detected on the ovary before being found in the liver. Hydatid cyst could theoretically present in this way, but there is no history of travel or exposure to suggest the patient is at risk for echinococcal infection. The clinical scenario is most consistent with a hepatic adenoma which typically occurs in women of reproductive age on oral contraceptive pills, and can sometimes become sufficient size to cause pain and hepatomegaly. CONCLUDING: With the evidence we have, the best hypothesis is that this woman's liver mass is a hepatic adenoma given the absence of other risk factors for the other conditions and corroborating with known risk factors like oral contraceptive use in the context of hepatic adenoma. RESPONSE: (A) Hepatic adenoma is the most likely diagnosis of this patient's liver mass considering her demographic, clinical and investigational findings.

**AI_B:** RESTATING THE QUESTION: You're asking me to diagnose a 34-year-old woman who's been experiencing a constant dull ache in her right upper abdominal quadrant for the last month. This discomfort is not associated with intake of food or defecation and does not disrupt her sleep. She's not reported any changes in appetite or bowel habits and occasional use of pain mitigating drugs like acetaminophen have provided her with some relief. Healthwise, she's been in good condition so far and is on contraceptives. Her vital signs are stable and typical, and there is a notable enlargement of her liver but no palpable growths or scarring indicative of cirrhosis have been noted. Liver function tests are normal and tests for hepatitis B and C have returned negative. An ultrasound has revealed a 4 centimeter mass in her right liver lobe. The diagnosis options include a liver adenoma, a primary liver cancer, a parasitic cyst, or cancer that has spread to the liver from the ovaries. PLANNING: There are multiple potential diagnoses under consideration here, each with its own assumptions. The presence of the mass could be indicative of hepatic adenoma, hepatocellular cancer, a hydatid cyst, or metastatic ovarian cancer. It's important to note that liver function tests were normal, implying a non-aggressive growth, and that no cirrhosis or masses were palpable. Long-term use of oral contraceptives can sometimes lead to liver adenomas, which would align with the benign indication of her liver function tests. CLINICAL REASONING: Given the absence of serum α-fetoprotein elevation, which is commonly elevated in hepatocellular carcinoma, and because she does not have a history of cirrhosis which is a common precursor to hepatocellular carcinoma, we might cast doubt on the hepatocellular carcinoma option. With regard to a hydatid cyst, it's a parasitic infection primarily transmitted through contact with infected dogs and a history is typically prevalent in certain geographic regions, which is not mentioned. Also, Hepatitis B and C, which could precede hepatocellular carcinoma, have been ruled out. With normal liver function tests, the assumption of metastatic ovarian cancer seems unlikely too, as one would expect abnormal liver function tests amidst such malignancy. CONCLUDING: After evaluating the given symptoms, history, and results from tests and examination, my diagnosis would lean towards a hepatic adenoma, which is a benign liver tumor that can form with long-term oral contraceptive use and can often present with hepatomegaly and symptoms like discomfort or pain but without significantly affecting liver function tests or serum α-fetoprotein concentrations. RESPONSE: (A) Hepatic adenoma is the most likely diagnosis.

**AI_E:** RESTATING THE QUESTION: A 34-year-old woman has been experiencing right upper quadrant abdominal pain and discomfort for a month. Her pain is not influenced by eating or bowel function, and it does not cause nausea or changes in appetite or bowel habits. It's continually present during the day but rarely disrupts her sleep. The current medication she takes is an oral contraceptive; other health factors appear normal. Physical examination of her abdomen has shown a larger than normal liver, but no detectable masses or signs of liver disease. Tests on her liver performance and level of serum α-fetoprotein are within normal bounds, and she tested negative for both hepatitis B and C. An abdomen ultrasound, however, has revealed a 4cm square mass in her right liver lobe. Four potential diagnoses are proposed: hepatic adenoma, hepatocellular cancer, a hydatid cyst, or metastatic ovarian cancer. The question is, which of these is most likely? PLANNING: To answer this, several assumptions need to be assessed. Firstly, that the abdominal pain is associated with the discovered hepatic mass; secondly, that the mass is the result of a pathological condition; thirdly, that this condition is one of the four proposed in the question; and fourthly, that the significant features of this case (the patient's age, gender, use of oral contraceptives, and the absence of other significant medical history or symptoms) have diagnostic significance. Given these assumptions, we must involve questioning if the patient's oral contraceptive usage might have influenced the development of the mass. We also have to entertain the possibility that the mass might not necessarily be malignant, as the patient lacks any systemic symptoms like weight loss, fever or anorexia. CLINICAL REASONING: Oral contraceptives are known to increase the risk of hepatic adenomas. Hepatic adenomas are benign liver tumors that tend to occur in young women who are taking oral contraceptives. This may be explained by the fact that estrogen stimulates the overgrowth of hepatocytes, which could lead to a mass in the liver. Furthermore, the patient's normal liver function tests and serum α-fetoprotein concentration could align more with a benign tumor like a hepatic adenoma than with a malignant tumor such as hepatocellular malignancy. The negative serologies for hepatitis B and C also make the hepatocellular carcinoma less likely. CONCLUDING: Based on her medical history and the features of her clinical presentation, the woman's disease likely has a link to the usage of oral contraceptives in her past. The benign nature of the tumor is suggested by both her lack of additional symptoms, as well as her normal liver function tests despite having a hepatic mass. RESPONSE: Given the patient's age, gender, use of oral contraceptives, and the absence of systemic symptoms or abnormal liver function tests or serum α-fetoprotein levels, the most likely diagnosis in this case is hepatitis adenoma (Option A). This is a benign liver tumor that is known to be associated with the use of oral contraceptives.

**AI_C:** RESTATING THE QUESTION: This scenario reflects a 34-year-old woman who is suffering from a persistent dull pain in her right upper quadrant since the past month. Her pain isn't affected by eating or defecating and doesn't impact her sleeping habits significantly. The pain can be occasionally relieved by acetaminophen, which is a pain reliever and fever reducer. Her health history is generally good with no remarkable past illnesses. The woman takes an oral contraceptive, which are pills that prevent pregnancy. Upon examining her, the doctor discovers her liver is enlarged, a condition known as hepatomegaly. The results of the liver function tests, serum α-fetoprotein concentration, and tests for hepatitis B and C came back normal. α-fetoprotein is a substance normally produced by a fetus's liver and yolk sac. In adults, high levels of AFP can be a sign of certain cancers. Through ultrasonography, a 4×4-cm mass is detected in the right lobe of the patient's liver. Based on the information, the medical professionals want to diagnose her health problem by choosing between a hepatic adenoma, hepatocellular cancer, a hydatid cyst, or metastatic ovarian cancer. PLANNING: The mentioned health conditions are quite distinctive in their presentation, prognosis, and treatment. Hepatic adenomas are benign liver tumours often associated with oral contraceptive use. Hepatocellular cancer (HCC) is a malignancy arising from hepatocytes, but her liver function tests and AFP levels are normal. A hydatid cyst is an infection caused by a tapeworm, an unlikely diagnosis with no symptoms of systemic infection. Metastatic ovarian cancer implies a primary tumour in the ovary, with secondary liver lesions. Given the absence of symptoms and signs associated with systemic malignancy, it may be unlikely. CLINICAL REASONING: Identifying the most fitting diagnosis from the four options could be challenging. Due to this, a thorough examination and rationalization of each choice are needed. It's important to take into account the duration, severity, the patient's health history, and evidence from tests and symptoms. For instance, taking the use of oral contraceptives into account, which is known to raise the risk of hepatic adenoma, chances of diagnosing her with hepatic adenoma would be higher. Other tests, including liver function tests and AFP concentration, also point away from hepatocellular cancer. CONCLUDING: Simultaneously, a metastatic ovarian cancer diagnosis would require proof of a primary ovarian tumour, which isn't mentioned in the patient's health and test results. Hydatid cysts, on the other hand, usually present with systemic infection symptoms, absent in this scenario. Thus, my conclusion tends toward a hepatic adenoma, as its correlation with oral contraceptive use seems to be the most likely fit in this case. RESPONSE: (A) Hepatic adenoma is the most likely diagnosis. The normal liver function tests and serum α-fetoprotein concentration, along with the absence of symptoms indicative of systemic malignancy or infection, make the other options less probable. The history of oral contraceptive use further signifies the likelihood of hepatic adenoma.

**Facilitator** The consensus statement synthesizing the responses of AI_A, AI_B, AI_C, AI_D, and AI_E is as follows: The clinical scenario presents a 34-year-old woman with persistent right upper quadrant abdominal pain, hepatomegaly, and a hepatic mass detected via ultrasound. Her medical history is unremarkable except for the use of oral contraceptives. Her liver function tests, serum α-fetoprotein concentration, and hepatitis B and C tests are all normal. The potential diagnoses considered are hepatic adenoma, hepatocellular cancer, hydatid cyst, and metastatic ovarian cancer. The patient's age, gender, use of oral contraceptives, and absence of other significant medical history or symptoms are key factors in the diagnostic process. Oral contraceptives are known to increase the risk of hepatic adenomas, benign liver tumors that tend to occur in young women. The normal liver function tests and serum α-fetoprotein concentration align more with a benign tumor like a hepatic adenoma than with a malignant tumor such as hepatocellular carcinoma. Hepatocellular carcinoma is less likely due to the absence of elevated α-fetoprotein levels, cirrhosis, and negative hepatitis B and C tests. A hydatid cyst, caused by a parasitic infection, is also unlikely due to the absence of exposure history and symptoms of systemic infection. Metastatic ovarian cancer is less probable due to the lack of accompanying symptoms such as menstrual irregularities, loss of appetite, or unexplained weight loss, and one would expect abnormal liver function tests amidst such malignancy. In conclusion, given the patient's demographic, clinical, and investigational findings, the most likely diagnosis is hepatic adenoma. This is supported by the patient's use of oral contraceptives, the benign nature of the tumor suggested by the lack of additional symptoms, and normal liver function tests and serum α-fetoprotein levels despite the presence of a hepatic mass.
